# Mental health and quality of life in patients with ruptured and unruptured cerebral arteriovenous malformation

**DOI:** 10.1055/s-0046-1820530

**Published:** 2026-05-12

**Authors:** Victor Santos Nascimento, Adriane Oliveira Lima, Amélia Muniz Pereira, Daniela de Souza Coelho, Marcos Devanir Silva da Costa, Raphael Wuo-Silva, Feres Chaddad-Neto

**Affiliations:** 1 Universidade Federal de São Paulo, Escola Paulista de Medicina, Departamento de Neurologia e Neurocirurgia, São Paulo SP, Brazil.; 2 Hospital Beneficência Portuguesa de São Paulo, Departamento de Neurocirurgia, São Paulo SP, Brazil.

**Keywords:** Intracranial Arteriovenous Malformations, Quality of Life, Mental Health, Cerebrovascular Disorders

## Abstract

**Background:**

Cerebral arteriovenous malformation (cAVM) is a rare and complex cerebrovascular disease that may cause neurological symptoms, cognitive complaints, and psychological distress even without hemorrhage, potentially impairing long-term quality of life.

**Objective:**

To assess the impact of cAVM on mental health and quality of life, considering patients' clinical presentation and the type of treatment received.

**Methods:**

In the present cross-sectional study, adults aged 18 to 65 with ruptured or unruptured cAVM treated with conservative, or multimodal approaches were recruited. Data were collected through an online survey distributed by participating institutions. Mental health, psychological distress, and quality of life were evaluated using the 5-item Mental Health Index (MHI-5), the Self-Reporting Questionnaire (SRQ-20), and the World Health Organization Quality of Life-brief version questionnaire (WHOQOL-BREF). Results were categorized and compared with normative data. Statistical analyses included descriptive statistics, analysis of variance (ANOVA), t-tests, χ ^2^ , and Fisher's exact tests.

**Results:**

Eighty-six participants with cAVM completed the questionnaire. Unruptured lesions accounted for 62.8% and ruptured cAVM 37.2%. Neurological deficits were reported by 94.2% of participants. Among conservatively-managed unruptured cases, 47.1% reported neurological deficits, without significant differences when compared with other treatments. Despite similar neurological status, conservatively managed unruptured patients showed poorer mental health ( *p* = 0.048) and lower quality of life in the physical ( *p* < 0.001) and social domains ( *p* = 0.016).

**Conclusion:**

Patients with unruptured cAVM managed conservatively exhibit significantly worse mental health and reduced physical and social quality of life, despite a similar prevalence of neurological deficits relative to other groups. These findings highlight the importance of incorporating psychosocial outcomes into treatment decision-making for cAVM.

## INTRODUCTION

Cerebral arteriovenous malformation (cAVM) is a rare and complex cerebrovascular lesion, with an estimated incidence of 1.12 to 1.34 per 100 thousand person-years. They are characterized by a nidus and early venous drainage, forming a direct shunt between arteries and veins without intervening capillaries. 1 2 3 The absence of capillaries exposes the veins to arterial pressure, increasing the risk of vessel rupture. 1 2 3 Intracranial hemorrhage (IH) is one of the main manifestations of cAVM, with an annual rupture risk of approximately 1 to 3% and a re-rupture rate of 7.6%. It is a serious condition that can lead to disability and death, and only 12 to 39% of survivors achieve functional independence. 4

Patients with cAVM, even before rupture, may present with neurological symptoms such as seizures, headaches, and dizziness. Cognitive changes can also occur, particularly affecting memory, language, visual perception, and executive functions, which may significantly impact quality of life. 5 6 7 8 9 Treatment of cAVM may be conservative or involve interventions such as endovascular embolization, surgery, radiosurgery, or multimodal therapeutic approaches. 10

Some studies have reported symptomatic improvement following treatment, particularly regarding cognitive deficits. 11 Therefore, selecting the most appropriate treatment should take into account the specific characteristics of each case and, above all, the prognosis associated with each therapeutic modality.

Given the limited literature on the psychological impact of cAVM and their effect on mental health and quality of life across different treatment modalities, the current study aims to evaluate how cAVM influences patients' mental health profiles and quality of life, considering both clinical presentation and the types of treatments received.

## METHODS

### Patients

The present cross-sectional observational study, conducted between 2021 and 2022, included patients aged 18 to 65 years with a confirmed diagnosis of ruptured or unruptured cAVM. The study was coordinated by Universidade Federal de São Paulo (Unifesp) in collaboration with Hospital Beneficência Portuguesa de São Paulo. Data were obtained through an online self-report survey distributed by the participating institutions, reaching patients from across Brazil.

Eligible patients had undergone conservative treatment, surgical resection, endovascular therapy, radiosurgery, or a multimodal approach. Patients with conditions likely to interfere with the outcome measures were excluded, including neurodevelopmental disorders, neurocognitive disorders, traumatic brain injury, brain tumors, and cerebral cavernous malformations.

Only patients who provided written informed consent participated in the study. The research was approved by the Research Ethics Committee of Unifesp and Hospital Beneficência Portuguesa de São Paulo, under protocol number 50297321.9.0000.5505.

### Data collection

We assessed patients' mental health, level of mental distress, and quality of life. The following instruments were used: 5-item Mental Health Index (MHI-5) to measure mental health, Self-Reporting Questionnaire (SRQ-20) to assess the level of mental distress, and World Health Organization Quality of Life-brief version questionnaire (WHOQOL-BREF) to assess the quality of life. 12 13 14 A structured questionnaire was also developed to collect socioeconomic data, lifestyle habits, symptoms, AVM characteristics, and treatment history. All forms were distributed via social media and in public settings, including bulletin boards and outpatient clinics.

The results were corrected and compared with the normative samples of the instruments. The MHI-5 scale is a brief version of the Short Form 36 instrument, assessing symptoms of anxiety and depression. It is scored from 0 to 100, and higher scores indicate better mental health. It can also be used to calculate the mental health index, for which a score ≤ 52 is internationally considered the cutoff for severe symptoms. 15 16 The SRQ-20 scale is the 20-item version of the SRQ-30 for tracking mental disorders, ranging from 0 to 20, with a higher score indicating a higher level of mental distress, increasing the likelihood of the presence of a mental disorder, with 8 points being the cutoff score. 13 The WHOQOL scale comprises 26 questions divided into four domains: physical, psychological, social relationships, and environment. Each item receives a score from 1 to 5, converted to a 0 to 100 scale, in which higher results indicate better quality of life. 14

The instruments were selected based on their widespread use in clinical and epidemiological research, particularly in studies evaluating psychological outcomes. Furthermore, priority was given to measures with proven cross-cultural validity and institutional endorsement, such as the WHOQOL-BREF, developed by the World Health Organization, and the SRQ-20, originally created by the same institution for screening mental disorders in various contexts. This ensured the use of brief, validated, and internationally comparable tools suitable for large-scale community data collection.

First, the data were analyzed using descriptive statistics, and then the Kolmogorov-Smirnov tests were performed to assess the data distribution and investigate evidence of normality, considering *p* < 0.05. One-way analysis of variance (ANOVA) and the T-test were performed to compare the sociodemographic results between the groups of ruptured and unruptured cAVM with the types of treatment.

We transformed the results of the scales into categorical information, which were classified as with or without the presence of mental distress, with or without quality of life, and with or without mental health. We used the χ2 test and Fisher's exact test when appropriate, considering *p* < 0.05. All statistical analyses were performed using the IBM SPSS Statistics for Windows (IBM Corp.), version 20.0.

## RESULTS

### Baseline characteristics

Eighty-six patients completed the online survey and were included, including 54 (62.8%) with unruptured cAVM and 32 (37.2%) with ruptured cAVM; no patients were excluded. The mean age was 36.16 ± 10.25 years. No statistically significant difference in age was found between the 2 groups (t(83) = -0.462, *p* = 0.645). Similarly, no age differences were observed with respect to treatment types (F(4) = 0.910, *p* = 0.462). Table 1 provides a more detailed description of the sociodemographic and clinical characteristics of the study participants.

**Table 1:** Sociodemographic and clinical data

Variable		Data
Mean age (years)	Ruptured arteriovenous malformation	35.50 ± 9.94
	Unruptured arteriovenous malformation	36.57 ± 10.51
	Total	36.16 ± 10.25
Level of schooling: n (%)	Elementary school	7 (8.1)
	High school	37 (43.0)
	Bachelor's degree	25 (29.1)
	Specialization	14 (16.3)
	Master's degree	1 (1.2)
	Doctor of Phylosophy	2 (2.3)
Arteriovenous malformation: n (%)	Ruptured	32 (37.2)
	Unruptured	54 (62.8)
Location: n (%)	Temporal	15 (17.4)
	Parietal	14 (16.3)
	Frontal	11 (12.8)
	Occipital	8 (9.3)
	Cerebellum	7 (8.1)
	Parietooccipital	3 (3.5)
	Frontoparietal	2 (2.3)
	Insula	1 (1.2)
	Corpus callosum	1 (1.2)
	Midbrain	1 (1.2)
	Mesencephalon	1 (1.2)
	Missing	22 (25.6)
Neurological symptoms: n (%)	Yes	81 (94.2)
	No	5 (5.8)
Treatment nr. (%)	Treated	57 (66.3)
	Untreated	29 (33.7)
Type of treatment: n (%)	Microsurgery	21 (24.4)
	Embolization	21 (24.4)
	Radiosurgery	6 (7.0)
	Multimodal	9 (10.5)

The participants in the present study had varied levels of schooling, with 43% (n = 37) having completed high school. Cerebral arteriovenous malformations were located in major brain regions, with the temporal lobe being the most frequent site (17.4%, n = 15).

**Figure 1:**
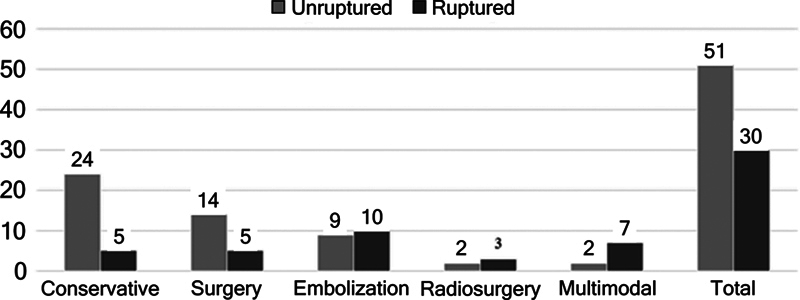
Frequency of neurological deficits according to type of treatment.

Neurological deficits were identified in 81 of the 86 participants (94.2%). Among these patients, 51 (63.0%) had unruptured cAVM and 30 (37.0%) had ruptured cAVM ( Figure 1 ).

In the unruptured cAVM group, neurological deficits were most frequently observed in patients managed conservatively, affecting 24 of 51 individuals (47.1%). In the ruptured cAVM group, neurological deficits were most common among patients treated with endovascular embolization (10 of 30; 33.0%), followed by those who underwent multimodal treatment (7 of 30; 23.4%) ( Figure 1 ).

Despite these descriptive differences, no statistically significant association was found between the presence of neurological deficits and treatment modality in either group (ruptured cAVM: χ ^2^ (4) = 3,685, *p* = 0.450; unruptured cAVM: χ ^2^ (4) = 3,494, *p* = 0.479).

### Mental health index

A statistically significant association was observed between poorer mental health and conservative treatment in patients with unruptured cAVM (χ ^2^ (4) = 8,994, *p* = 0.048). Among participants classified as having poorer mental health, 46 of 70 patients (65.7%) belonged to the unruptured cAVM group ( Table 2 ).

**Table 2:** Assessment results of mental health (MHI-5and mental suffering) and quality of life scales

Index	Treatment	N	Unruptured AVM: n (%)	Ruptured AVM: n (%)	Total sample: n (%)
MHI-5	Conservative	29	21 (80.8)	5 (19.2)	26 (100)
	Surgery	21	12 (75.0)	4 (25.0)	16 (100)
	Embolization	21	9 (52.9)	8 (47.1)	17 (100)
	Radiosurgery	6	2 (50.0)	2 (50.0)	4 (100)
	Multimodal	9	2 (28.6)	5 (71.4)	7 (100)
	Total	86	46 (65.7)	24 (34.3)	70 (100)
Mental suffering	Conservative	29	14 (73.7)	5 (26.3)	19 (100)
	Surgery	21	7 (70.0)	3 (30.0)	10 (100)
	Embolization	21	9 (60.0)	6 (40.0)	15 (100)
	Radiosurgery	6	1 (50.0)	1 (50.0)	2 (100)
	Multimodal	9	1 (16.7)	5 (83.3)	6 (100)
	Total	86	32 (61.5)	20 (38.5)	52 (100)
WHOQOL Physical	Conservative	29	17 (94.4)	1 (5.6)	18 (100)
	Surgery	21	13 (76.5)	4 (23.5)	16 (100)
	Embolization	21	5 (41.7)	7 (58.3)	12 (100)
	Radiosurgery	6	0 (0.0)	4 (100.0)	4 (100)
	Multimodal	9	2 (33.3)	4 (66.7)	6 (100)
	Total	86	37 (64.9)	20 (35.1)	57 (100)
WHOQOL Psychological	Conservative	29	9 (64.3)	5 (35.7)	14 (100)
	Surgery	21	7 (63.6)	4 (36.4)	11 (100)
	Embolization	21	6 (46.2)	7 (53.8)	13 (100)
	Radiosurgery	6	2 (66.7)	1 (33.3)	3 (100)
	Multimodal	9	2 (33.3)	4 (66.7)	6 (100)
	Total	86	26 (55.3)	21 (44.7)	47 (100)
WHOQOL Social Relationship	Conservative	29	12 (70.6)	5 (29.4)	17 (100)
	Surgery	21	5 (62.5)	3 (37.5)	8 (100)
	Embolization	21	6 (40.0)	9 (60.0)	15 (100)
	Radiosurgery	6	1 (100.0)	0 (0.0)	1 (100)
	Multimodal	9	0 (0.0)	6 (100.0)	6 (100)
	Total	86	24 (51.1)	23 (48.9)	47 (100)
WHOQOL Environment	Conservative	29	7 (63.6)	4 (36.4)	11 (100)
	Surgery	21	3 (50.0)	3 (50.0)	6 (100)
	Embolization	21	6 (60.0)	4 (40.0)	10 (100)
	Multimodal	9	0 (0.0)	4 (100)	4 (100)
	Total	86	16 (51.6)	15 (48.4)	31 (100)

**Abbreviations** : AVM, arteriovenous malformation; MHI-5, 5-item Mental Health Index; WHOQOL, World Health Organization Quality of Life.

In patients with ruptured cAVM, no statistically significant association was found between mental health status and treatment modality. However, within this group, poorer mental health was most frequently observed in patients treated with endovascular embolization, accounting for 8 of 17 cases (47.1%).

### Self-reporting questionnaire

Among patients with unruptured cAVM who met criteria for psychological distress, 14 of 32 individuals (43.8%) had received conservative treatment. In contrast, among patients with ruptured cAVM and psychological distress, endovascular embolization was the most frequent treatment modality, observed in 6 of 20 patients (30.0%).

Overall, psychological distress was more frequent in the unruptured cAVM group (32 of 52 patients; 61.5%) than in the ruptured cAVM group (20 of 52 patients; 38.5%). No statistically significant associations were identified between psychological distress and treatment modality in either group.

### WHOQOL – quality of life

A significant association was observed between unruptured cAVM managed conservatively and poorer quality of life in the physical domain (χ ^2^ (4) = 20,586, *p* < 0.001). Similarly, conservative treatment in unruptured cAVM was significantly associated with worse quality of life in the social relationships domain (χ ^2^ (4) = 10,908, *p* = 0.016).

**Figure 2:**
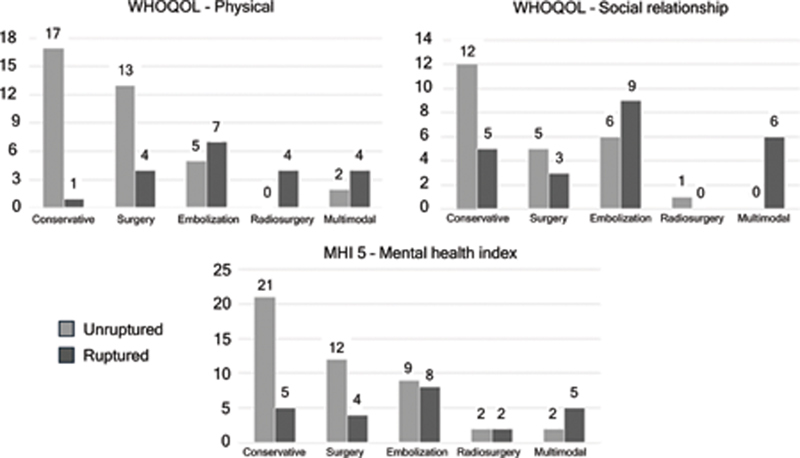
Comparison of scale scores by treatment type. Across all three indices, patients with unruptured arteriovenous malformation and conservative treatment were associated with poorer quality of life and worse mental health outcomes. Results were considered statistically consistent when *p* < 0.05.

In the psychological domain, among patients with low quality of life, 7 of 21 individuals (33.3%) in the ruptured cAVM group had undergone endovascular embolization. In the unruptured cAVM group, 9 of 26 patients (34.6%) with reduced psychological quality of life had received conservative treatment. Overall, more than half of the patients with impaired psychological quality of life belonged to the unruptured cAVM group (26 of 47; 55.3%) ( Figure 2 ). No additional statistically significant associations were observed in this domain.

Regarding the environmental domain, patients with ruptured cAVM treated conservatively, by endovascular embolization or through multimodal therapy, exhibited the same proportion of reduced quality of life (4 of 15 patients in each group; 26.7%). Among patients with unruptured cAVM and reduced environmental quality of life, conservative treatment was the most frequent modality, accounting for 7 of 16 cases (43.8%) ( Table 2 ).

## DISCUSSION

In the present study, we found that among patients with unruptured cAVM and conservative medical management, 45.7% of patients presented symptoms of anxiety and depression, which impacted the mental health index compared to the healthy population. In addition, patients also presented a reduction in aspects of quality of life, which may represent a compromise in the comprehensive concept of health established by the World Health Organization (WHO). 17 According to the WHO, it is estimated that 4.4% of the world's population has symptoms of anxiety and, in the same proportion, symptoms of depression, both of which can directly impact people's quality of life. 18

Cerebral arteriovenous malformation is a complex cerebrovascular condition that may lead to neurological and cognitive impairments, factors closely linked to diminished quality of life. Despite this, the main studies in the field have not focused on understanding how AVMs affect quality of life or how these factors influence treatment decision-making. Notably, the leading randomized clinical trial addressing AVM treatment did not incorporate measures of cognitive functioning, mental health, or quality of life. 19

Considering these gaps, the present study is the first to investigate aspects of mental health and quality of life in a sample of cAVM patients from Brazil. Studies in other populations have evaluated quality of life, mental health, and depressive symptoms in patients with cAVM, considering variations in treatments and outcomes. Our results align with some previous findings but diverge from others. For example, the study by van der Schaaf et al. 20 found no difference in quality of life between patients with and without intracranial hemorrhage due to cAVM. In contrast, our study identified a worse quality of life associated with conservative treatment of unruptured cAVM. Regarding psychological status, they showed that knowledge of the presence of an untreated neurological disease could reduce quality of life, especially in psychosocial domains, which may also impact mental health, both due to the experience of living with an untreated disease and due to the waiting period before potential treatment.

The discrepancies between the findings of van der Schaaf et al. 20 and those of our study may be attributed to differences in sample composition, as their study included 9 patients with cerebral aneurysms and 12 with cAVM. Furthermore, they assessed quality of life using the Sickness Impact Profile (SIP), which differs from the instruments applied in our study, and assessed aspects of anxiety and depression with the Hospital Anxiety and Depression Scale (HADS), which is an instrument adapted only for the hospital environment, unlike our instrument that can also be used in clinical environments.

In the present study, 94.2% of patients had neurological symptoms, such as hemiparesis, headache, or epileptic seizure, and 35.8% were treated conservatively. The study carried out by Pohjola et al., 21 with 262 participants, also reported a relationship between multiple episodes of bleeding or refractory epilepsy and a significantly impaired quality of life. Neurological deficits can negatively affect quality of life through cognitive difficulties, mobility limitations, and visual impairments. These factors may help explain why patients in our study, who were treated conservatively, presented worse quality of life, especially in the domains related to physical aspects and social relationships.

The study conducted by Orosz et al. 22 with 36 patients found results congruent with ours, demonstrating that patients with untreated and unruptured cAVM experienced significant impacts on quality of life. In their work, the worsening quality of life was associated with symptoms of anxiety, depression, pain, and discomfort. Benhassine et al. 23 described in a study with 73 patients (42 with ruptured cAVM and 31 with unruptured cAVM) a strong correlation between mental health and quality of life in patients with untreated cAVM. Of the 31 patients with unruptured cAVM, 28 presented some level of depressive symptoms, and 48.38% of these patients received conservative management. The research carried out by O'Donnell et al. 24 with 45 participants, 35 of whom had unruptured cAVM, supports these findings, reporting an improvement in quality of life 12 months after surgical resection of the cAVM.

Although no statistically significant association was found between mental distress and the different groups, 63.41% of the patients exhibited a high level of mental distress, and patients with conservative treatment in the unruptured cAVM group presented more significant mental distress (43.8%), which was also seen in the result of the mental health index. Overall, these results indicate that patients with cAVM generally experience higher levels of mental distress and worse mental health compared to the general population. These findings highlight the need for closer attention and support for patients with unruptured cAVM.

The A Randomized trial of Unruptured Brain Arteriovenous malformations (ARUBA) study suggests that conservative treatment is superior to surgical treatment in terms of morbidity and mortality. 19 However, this study does not consider these patients' psychological aspects, mental health, and quality of life. On the other hand, our findings and the existing literature indicate that psychological aspects, mental health, and quality of life should be considered when deciding on treatment. Living with a neurological disease with a risk of IH can significantly impact the daily lives of patients. Interventional treatments, such as surgery, can play an important role in improving quality of life, and further studies considering mental health and quality of life outcomes are needed.

Despite its contributions, the current study had some limitations. Online data collection may have influenced participants' responses due to potential difficulties in understanding the questions or distractions during completion. This approach also limited the collection of more technical clinical information, such as detailed characteristics of cAVMs and standardized grading scales (e.g., the Spetzler-Martin scale), and may have increased the risk of selection bias, particularly favoring the inclusion of symptomatic patients. Additionally, the use of certain medications may have affected cognitive performance and, consequently, quality-of-life outcomes. Furthermore, the inclusion of patients from different geographic regions reduced control over the consistency and quality of medical communication regarding the disease, making it difficult to evaluate how this factor influenced patients' perceptions and understanding of their condition. Nevertheless, although causal relationships cannot be established, the findings indicate the presence of correlations between mental health outcomes and cAVM; however, larger studies employing different study designs are needed to confirm these findings.

In conclusion, in the present cross-sectional study, patients with unruptured cAVMs treated conservatively showed an association with poorer mental health and lower quality of life, particularly in the physical and social domains. These findings should be interpreted with caution and do not establish causality. However, they highlight the importance of considering mental health and quality of life in this population. Further studies are needed to better characterize these issues and support clinical decision-making in the management of patients with cAVM.
